# Identification and Characterization of *Hyphantria cunea* Aminopeptidase N as a Binding Protein of *Bacillus thuringiensis* Cry1Ab35 Toxin

**DOI:** 10.3390/ijms18122575

**Published:** 2017-11-30

**Authors:** Yakun Zhang, Dan Zhao, Xiaoping Yan, Wei Guo, Yajun Bao, Wei Wang, Xiaoyun Wang

**Affiliations:** 1 College of Plant Protection, Hebei Agricultural University, Baoding 071000, China; yakun_0830@126.com (Y.Z.); zhaodan@hebau.edu.cn (D.Z.); yxp890619@163.com (X.Y.); 15732233196@163.com (Y.B.); wwjiayou85@126.com (W.W.); 2 Plant Science and Technology College, Beijing University of Agriculture, Beijing 102206, China; 3 College of Agriculture, Northeast Agricultural University, Harbin 150038, China; wxy3236@126.com

**Keywords:** *Hyphantria cunea*, HcAPN3, Cry1Ab, binding protein, RNAi

## Abstract

The fall webworm, *Hyphantria cunea* (Drury) is a major invasive pest in China. Aminopeptidase N (APN) isoforms in lepidopteran larvae midguts are known for their involvement in the mode of action of insecticidal crystal (Cry) proteins from *Bacillus thuringiensis*. In the present work, we identified a putative Cry1Ab toxin-binding protein, an APN isoform designated HcAPN3, in the midgut of *H. cunea* by ligand blot and mass spectrometry. *HcAPN3* was highly expressed throughout all larval developmental stages and was abundant in the midgut and hindgut tissues. *HcAPN3* was down-regulated at 6 h, then was up-regulated significantly at 12 h and 24 h after Cry1Ab toxin treatment. We expressed HcAPN3 in insect cells and detected its interaction with Cry1Ab toxin by ligand blot assays. Furthermore, RNA interference (RNAi) against *HcAPN3* using oral delivery and injection of double-stranded RNA (dsRNA) resulted in a 61–66% decrease in transcript level. Down-regulating of the expression of HcAPN3 was closely associated with reduced susceptibility of *H. cunea* to Cry1Ab. In addition, the HcAPN3E fragment peptide expressed in *Escherichia coli* enhanced Cry1Ab toxicity against *H. cunea* larvae. This work represents the first evidence to suggest that an APN in *H. cunea* is a putative binding protein involved in Cry1Ab susceptibility.

## 1. Introduction

The fall webworm, *Hyphantria cunea* (Drury) (Lepidoptera: Arctiidae) is a severe economic pest that originated in North America [1]. Since it was first recorded in China in 1979, *H. cunea* has widely expanded its distribution to more regions in eastern and northeastern China, such as Shandong, Hebei, Shanxi, Jiangsu, Henan and Anhui provinces [2,3]. As reported, the occurrence of *H. cunea* in China increased by 20.9% when compared with the previous year [4]. *H. cunea* has caused significant ecological damage and economic losses in forests, fruit trees and agricultural crops in China due to its extremely broad host range [5,6].

Aminopeptidase N (APN) family is composed of a class of zinc metalloproteinases that preferentially cleave single neutral amino acids from the N-terminus of polypeptides [7]. They are distributed widely in the plant and animal kingdoms and highly expressed in the brush border membranes of the alimentary tract in lepidopteran larvae [8,9]. APNs are involved in several functions in a wide range of species; in the lepidopteran larval midgut they play an important role in protein digestion, co-operating with endopeptidases and carboxypeptidases to digest proteins derived from the diet [10]. The typical features present in classical lepidopteran APNs includes a potential signal peptide at the N-terminus, a characteristic zinc-binding motif HEXXH(X)_18_E essential for their enzymatic activity, a highly conserved GAMEN motif forming part of the active site and a glycosylphosphatidylinositol (GPI) anchor signal sequence at the C-terminal attaching them to the membrane [7,11]. APNs are encoded in insects by the members of a multi-gene family [12,13]. To date, at least eight different clusters of APNs have been defined in Lepidoptera (APN1-8) [12]. Each lepidopteran species shows no more than one sequence for each cluster with the exception of Class 3, which includes more isoforms in several species [12,14].

In addition to being studied for their role in digestion, APNs in insects have been extensively investigated for their interactions with *Bacillus thuringiensis* (*B. turingiensis*, Bt) crystal insecticidal (Cry) toxins [15]. So far, APN [7], cadherin [16], alkaline phosphatase (ALP) [17] and ATP-binding cassette transporter subfamily C protein (ABCC) [18,19] have been well studied as Cry toxin receptors. Since an APN was first proposed as a Cry receptor in *Manduca sexta* [20], increasing numbers of APNs have been isolated and characterized from the midgut of lepidopterans and their involvement in Cry susceptibility have been proved [7,14,21,22]. Alteration of APNs in the midgut brush border membrane vesicles (BBMV) have been proposed to be responsible for reducing or increasing Bt resistance of insects. For example, in *Spodoptera exigua*, the absence of expression of APN1 causes resistance to Cry1Ca [23], a deletion mutation of HaAPN1 is associated with *Helicoverpa armigera* resistance to Cry1Ac toxin [24], and the resistance to Cry1Ac in the cabbage looper, *Trichoplusia ni*, is associated with differential alteration of midgut APN1 and APN6, conferred by a trans-regulatory mechanism [25].

Cry1Ab toxin exhibits high toxicity against various lepidopteran larvae and it has been used in transgenic corn and rice to control *Ostrinia nubilalis* Hübner and *Chilo suppressalis* worldwide [26,27]. The binding proteins or receptors involved in Cry1Ab susceptibility from different lepidopteran species have been isolated or identified. In *Bombyx mori*, a cadherin-like protein BtR175 and BmAPN1 were found to bind Cry1Ab [28]; linkage analysis revealed that Cry1Ab resistance in *B. mori* was linked to alterations in an ABCC2 protein [19,29]. Two *Manduca sexta* GPI-anchored proteins, APN and ALP, were shown to bind Cry1Ab by ligand blot analysis [17,30]. A 220 kDa cadherin-like protein, OnBt-R(1), was identified as a Cry1Ab receptor of *O. nubilali*, and binding analysis showed that the cell-expressed OnAPN1 interacted with Cry1Ab [22,31]. Moreover, using stably expressing non-lytic clonal Sf9 cell lines expressing either HevABCC2 or HevCaLP of *Heliothis virescens* or both together confirmed that HevABCC2 was the central target in Cry1Ab toxin mode of action, and that HevCaLP played a supporting role in increasing Cry1Ab toxicity [18]. Furthermore, Cry1Ab binding protein APNs in the midgut of *C. suppressalis* were identified by ligand blot and mass spectrometry, and down-regulating expression of CsAPNs by RNAi was closely associated with reduced susceptibility of *C. suppressalis* to Cry1Ab [14].

In our previous work, a novel Cry1A toxin, Cry1Ab35 (Accession number KT01852882), with high insecticidal activity to *H. cunea* was isolated and expressed in the *B. thuringiensis* acrystalliferous mutant HD73^−^. In this study, The Cry1Ab binding protein, designated HcAPN3, in the midgut of *H. cunea* was identified by ligand blot and mass spectrometry. Its spatiotemporal expression and the Cry1Ab-induced expression profiles were investigated. Furthermore, the role of HcAPN3 in Cry1Ab susceptibility was supported by RNA interference (RNAi). Due to the crucial role of toxin binding proteins or receptors in Bt susceptibility [32], the identification of Cry1Ab interaction proteins is essential for better understanding of Bt toxicity mechanisms and developing effective strategies for pest control.

## 2. Results

### 2.1. Toxicity of Cry1Ab35 Toxin against H. cunea

**Figure 1:**
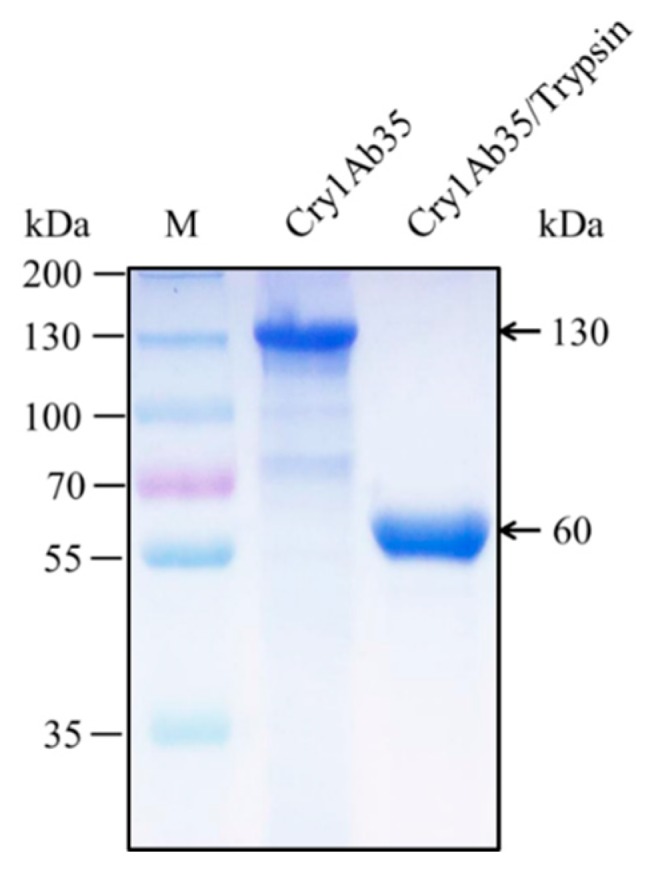
SDS-PAGE (10%) analysis of Cry1Ab35 expressed in *B. thuringiensis* HD73^−^ strain and the activated Cry1Ab35 toxin digested by trypsin. Lane M shows molecular weight markers.

Cry1Ab35 was expressed as a 130 kDa protein in *B. thuringiensis* acrystalliferous mutant HD73^−^ and yielded a 60 kDa fragment upon 1/20 (*w*/*w*) trypsin activation (Figure 1). The Cry1Ab35 toxin exhibited high toxicity against *H. cunea* first instar larvae and the LC_50_ was 4.34 μg/mL (slope ± SEM, 0.90 ± 0.14; chi-square, 4.25; 95% confidence interval, 1.96–12.27).

### 2.2. Detection of Cry1Ab-Binding Proteins in H. cunea BBMV

**Figure 2:**
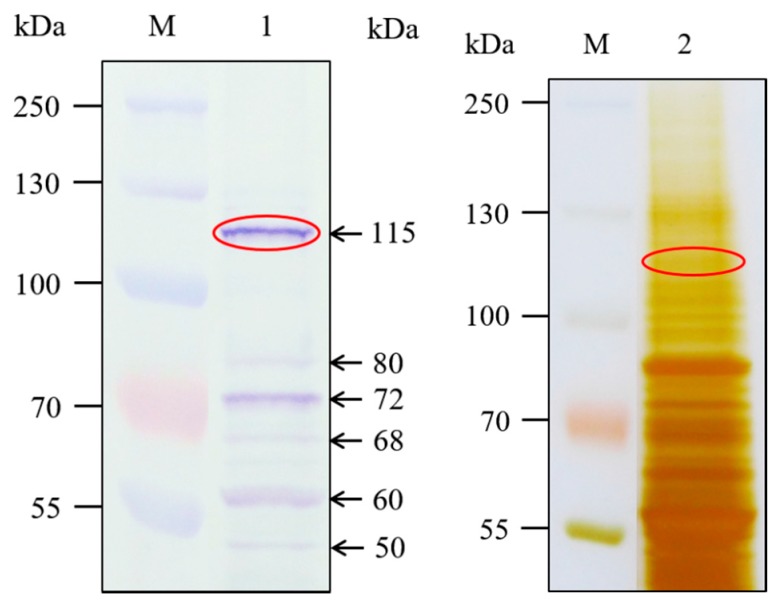
Ligand blot assay of *H. cunea* BBMV proteins (20 µg) immunodetected with anti-Cry1Ab35 antibodies. Lane M shows molecular weight markers. Ligand blot conducted with Cry1Ab35 toxin is shown in lane 1. SDS-PAGE (10%) profile of *H. cunea* BBMV proteins is shown in lane 2. Ellipses indicate SDS-PAGE band excised and analyzed by LC-MS/MS.

A ligand blot assay with the Cry1Ab35 toxin was performed to obtain the Cry1Ab-binding proteins in *H. cunea* fifth instars BBMV. The result showed that the protein band displaying the highest relative Cry1Ab binding intensity was approximately 115 kDa, with several smaller protein bands appearing at relative lower intensity (Figure 2, lane 1). The 115 kDa band was excised from the SDS-PAGE gel (Figure 2, lane 2) and analyzed by LC-MS/MS. Candidate proteins identified in the 115 kDa band were ranked by protein Qscore [33] (Table 1). Database searches with the peptides recovered from the 115 kDa band revealed the presence of APN isoforms with relative higher protein Qscore among candidate proteins (*p* < 0.05) (Table 1). In the present work, our attention was focused on the APN isoform 3 in *H. cunea*, which was identified as the most likely Cry1Ab binding protein through peptide alignment analysis.

### 2.3. Cloning and Characterization of H. cunea HcAPN3

PCR with primers designed from two APN peptide sequences yielded a 922 bp amplicon. Additional partial sequences were obtained by 5′RACE (1.4 kb) and 3′RACE (1.7 kb) PCR reactions. The full-length open reading frame (ORF) of *HcAPN3* was 2895 bp, which encoded a 108.2 kDa protein composed of 952 amino acids. According to the BLAST result, the gene exhibited the highest identity with *LdAPN3* from *Lymantria dispar* and was designated *HcAPN3* (Accession number KJ013598).

**Figure 3:**
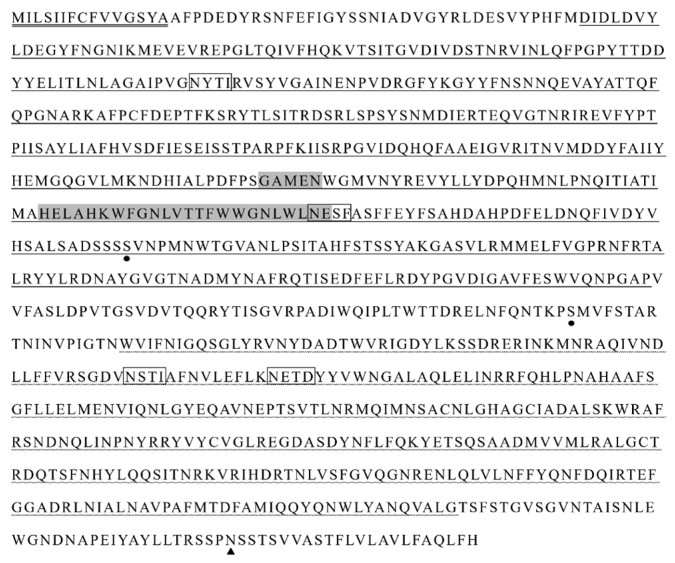
In silico analysis of the putative HcAPN3 protein. The putative N-terminal signal peptide is double-underlined and GPI anchor cleavage site is indicated by black triangle. The GAMEN and HEXXH(X)_18_E zinc-binding site motifs are in shadow. The predicted *O*-glycosylated residues are indicated by black dots and the putative *N*-glycosylation sites are boxed. Two conserved domains, gluzincin aminopeptidase domain and ERAP1-like C-terminal domain are underlined and dash-underlined, respectively.

**Figure 4:**
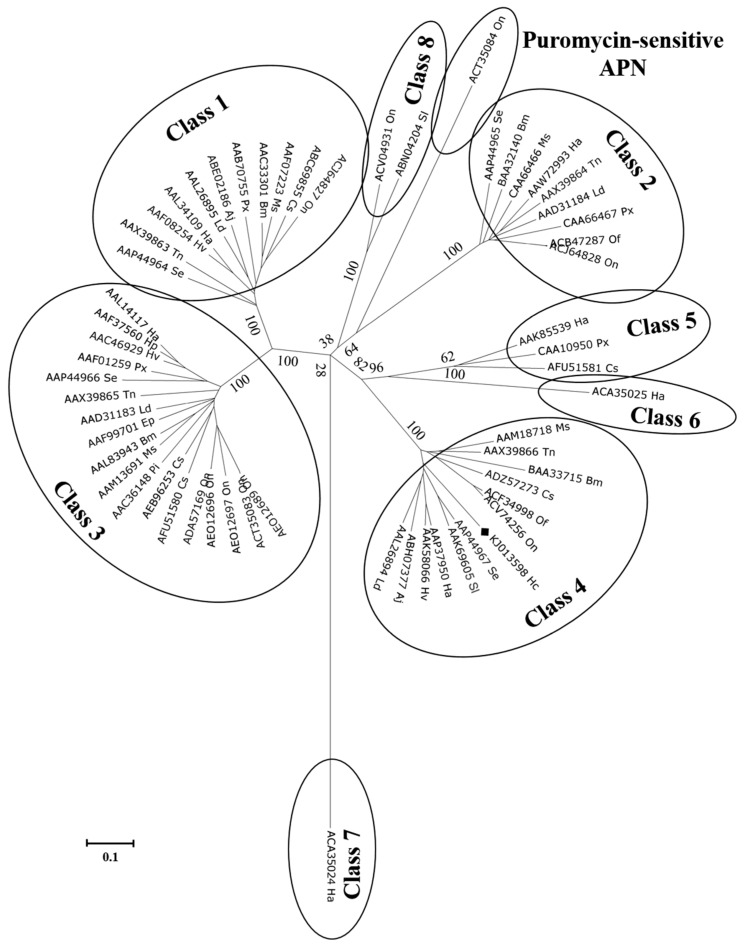
Phylogenetic tree derived from Clustal X alignment of complete lepidopteran APN protein sequences deposited in GenBank. Bootstrap values, expressed as percentages of 1000 replicates, are indicated for the principal nodes. The accession number of each amino acid sequence are indicated. Species abbreviations are as follows: Hc: *Hyphantria cunea*, Se: *Spodoptera exigua*, Tn: *Trichoplusia ni*, Ha: *Helicoverpa armigera*, Hp: *Helicoverpa punctigera*, Hv: *Heliothis virescens*, Ld: *Lymantria dispar*, Aj: *Achea janata*, Px: *Plutella xylostella*, Pi: *Plodia interpunctella*, Bm: *Bombyx mori*, Ms: *Manduca sexta* Cs, *Chilo suppressalis*, On: *Ostrinia nubilalis*, Of: *Ostrinia furnacalis*, Sl: *Spodoptera litura*, Ep: *Epiphas postvittiana*.

In silico analysis of the putative HcAPN3 protein identified APN typical motifs in homologous positions (Figure 3): a 15-amino acid signal peptide in the N-terminus, the GAMEN gluzincin aminopeptidase sequence and the zincbinding motif HEXXH(X)_18_E, a GPI-anchor site in the C-terminus. Moreover, it exhibited four predicted *N*-glycosylation sites and two potential *O*-glycosylation sites. It contained two conserverd domains, gluzincin aminopeptidase domain and ERAP1-like C-terminal domain. Phylogenetic analysis of lepidopteran complete APN sequences deposited in GenBank resulted in nine clusters (Figure 4). HcAPN3 was clustered in Class 4 APN.

### 2.4. Expression of HcAPN3G and HcAPN3E and Their Binding to Cry1Ab35 Toxin

**Figure 5:**
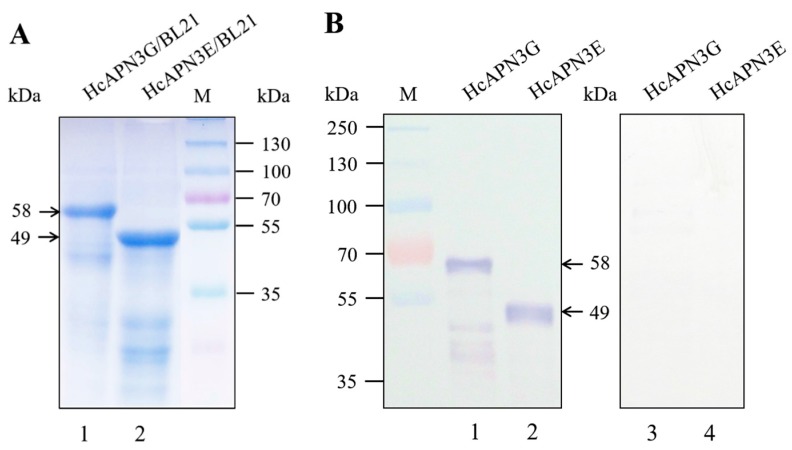
HcAPN3G and HcAPN3E fragment peptides expressed in *E.coli* BL21 (DE3) cells. HcAPN3G (lane 1) and HcAPN3E (lane 2) peptides expressed in *E. coli* BL21 (DE3) cells were separated by 10% SDS-PAGE, and either stained with Coomassie Brilliant Blue R-250 (**A**) or transferred to a PVDF membrane and probed by Cry1Ab35 toxin in ligand blot assay (**B**) with anti-Cry1Ab35 antibodies. Lane 3 and 4 was HcAPN3G and HcAPN3E control without Cry1Ab35 toxin ligand respectivily. Lane M shows molecular weight markers and arrows indicate the 58 kDa HcAPN3G and 49 kDa HcAPN3E protein bands.

SDS-PAGE analysis showed that HcAPN3G and HcAPN3E fragment peptides were expressed as a 58 and 49 kDa ptotein corresponding to the predicted molecular weights respectively in *E. coli* BL21 (DE3) cells (Figure 5A). Moreover, the binding of Cry1Ab35 toxin to HcAPN3G and HcAPN3E was determined by ligand blot analysis (Figure 5B).

### 2.5. Binding of Cry1Ab35 Toxin to HcAPN3 Expressed in H. cunea BBMV and Infected Sf9 Cells

**Figure 6:**
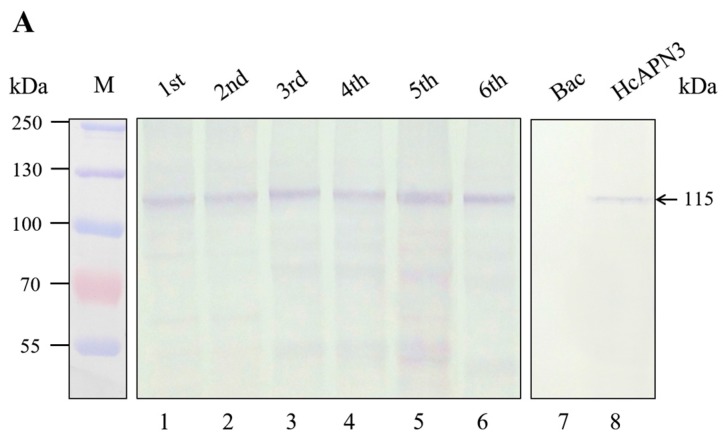

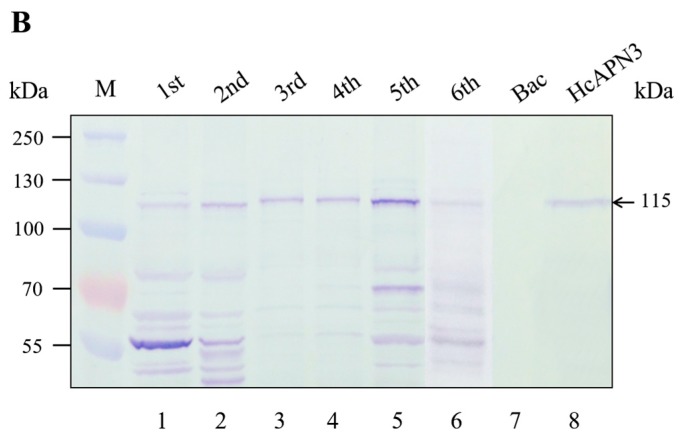
Detection and characterization of HcAPN3 expressed in *H. cunea* BBMV from different development larval stages and infected Sf9 cells. Protein from BBMV prepared from different developmental instars (lane 1–5), Sf9 cells containing control baculovirus alone (lane 6) and Sf9 cells infected with recombinant baculoviruse (lane 7) were separated by 10% SDS-PAGE and electrotransferred to PVDF membrane. (**A**) Western blot analysis of the expression of HcAPN3 with anti-HcAPN3 antibodies; (**B**) Binding detection of Cry1Ab35 toxin to HcAPN3 by ligand blot assay with anti-Cry1Ab35 antibodies. Lane M shows molecular weight markers and arrows indicate the position of the HcAPN3 protein in *H. cunea* BBMV.

Using the anti-HcAPN3 antibodies, we detected an immunoreactive band of 115 kDa in *H. cunea* BBMV from different development larval stages (Figure 6A, lane 1–6) as well as in Sf9 cells infected with recombinant baculovirus containing full-length *HcAPN3* (Figure 6A, lane 8). The protein was not detected in cells infected with vector alone (Figure 6A, lane 7).

Ligand blot assays showed that an approximately 115 kDa band corresponding to the HcAPN3 protein was probed by Cry1Ab35 toxin in *H. cunea* BBMV from different developmental stages (Figure 6B, lane 1–6) as well as in infected Sf9 cells (Figure 6B, lane 8). The binding protein corresponding to HcAPN3 detected in BBMV from the first and second instars was slightly smaller than that of third, fourth, fifth, and sixth instars (Figure 6B, lane 1,2). In the sixth instar larvae, the 115 kDa binding band was rarely detected (Figure 6B, lane 6). It is worth noting that a 58 kDa band detected in BBMV from the first and second instar larvae represented relative higher intensity than the 115 kDa binding band (Figure 6B, lane 1,2). Moreover, several smaller protein bands appearing with relative weaker binding intensity were detected in *H. cunea* BBMV from different larval stages (Figure 6B, lane 1–6).

### 2.6. Spatiotemporal and Induced Expression Profiles of HcAPN3

**Figure 7:**
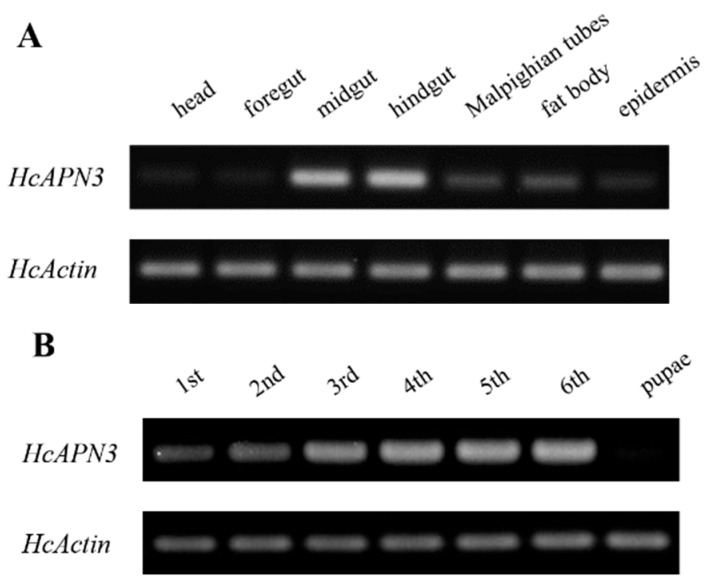

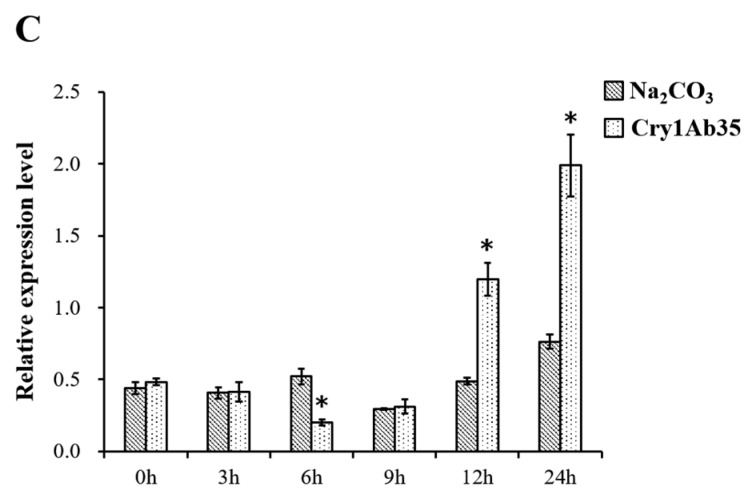
Expression profiles of *HcAPN3* in different larval tissues (**A**); in different developmental stages (**B**) and induced by Cry1Ab35 toxin (**C**). Tissue and temporal expression of *HcAPN3* were analyzed by semi-quantitative PCR. Tissues were isolated from fifth instar larvae including the foregut, midgut, hindgut, fat body, Malpighian tubules, and epidermis. Larval developmental stages include the first to sixth instars and pupae. Induced expression profile of *HcAPN3* with dietary Cry1Ab35 toxin was measured by qPCR. Larvae fed 50 mM Na_2_CO_3_ (pH 10.0) were used as the negative control. RNA of midgut tissues was extracted at 0, 3, 6, 9, 12, and 24 h after treatment. The housekeeping gene *HcActin* was shown as the internal control and the experiments were performed in three biological replicates. The mean ± standard errors (±SEM) was calculated to measure the relative expression levels by the 2^−ΔΔ*C*t^ method. Statistically significant differences from control samples are indicated by asterisks (*p* < 0.05, SNK test).

The spatiotemporal expression profiles of *HcAPN3* in different larval tissues and developmental stages were analyzed by the semi-quantitative PCR. Tissue-specific expression analysis revealed that *HcAPN3* was highly abundant in the midgut and hindgut, when compared with the Malpighian tubules and fat body tissues. However, it decreased to a very low expression level in the foregut, head and epider tissues (Figure 7A). Developmental expression results showed that *HcAPN3* was expressed throughout the entire larval cycle and highly expressed in the third, fourth, fifth, and sixth instars. In contrast, it was scarcely detected in pupae (Figure 7B).

The expression pattern of *HcAPN3* induced by dietary Cry1Ab35 toxin was investigated by qPCR. The result revealed that the gene was down-regulated at 6 h (*p* < 0.05, SNK test), then was up-regulated significantly at 12 and 24 h time points after Cry1Ab35 treatment (*p* < 0.05, SNK test) (Figure 7C).

### 2.7. Silencing of HcAPN3 Expression by RNAi

**Figure 8:**
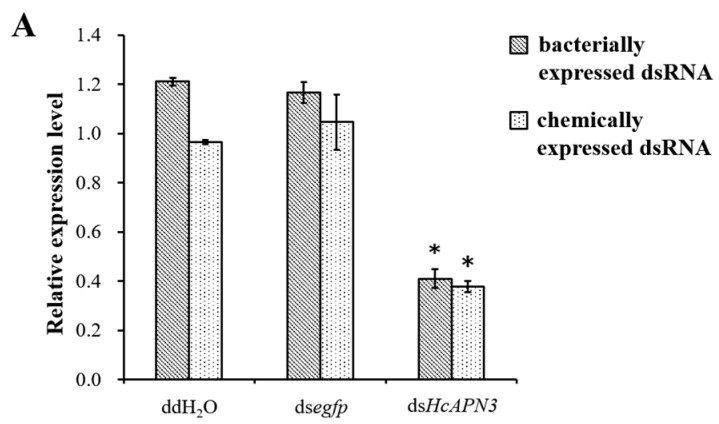

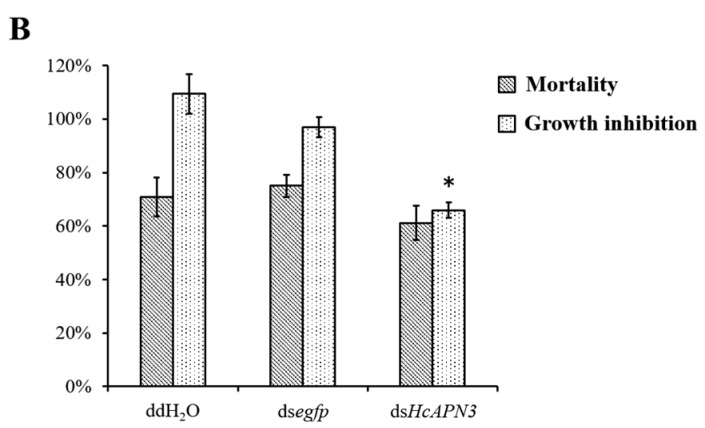
(**A**) Silencing of *HcAPN3* by feeding bacterially expressed dsRNA or injection of chemically synthesized dsRNA to *H. cunea* larvae and (**B**) effects of *HcAPN3* silencing on the toxicity of Cry1Ab35 toxins. (**A**) The relative expression levels of *HcAPN3* were determined by qPCR relative to the *HcActin* housekeeping gene. Non-silenced larvae treated with ddH_2_O and silenced larvae treated with *egfp* dsRNA were shown as controls. The data were calculated by 2^−ΔΔ*C*t^ method and presented as means ± SEM with three biological replicates; (**B**) Mortality and growth inhibition of *HcAPN3*-silenced larvae, non-silenced (ddH_2_O) larvae and *egfp*-silenced control larvae were determined after exposure to a diet containing 4 µg/mL Cry1Ab35 toxin for 4 days. Asterisks indicate significant differences (*p* < 0.05, SNK test).

Bacterially expressed dsRNA targeting *HcAPN3* were dietarily introduced into *H. cunea* first instar larvae. After continuous ingestion of dsRNA for 4 days, the transcript level of *HcAPN3* in larvae was reduced by approximately 66%, which was significantly lower compared to that in the larvae feeding on double-distilled water (ddH_2_O) or *egfp* dsRNA-overlaid diet (*p* < 0.05, SNK test) (Figure 8A). We did not detect significant differences in Cry1Ab35-induced mortality in HcAPN3 dsRNA-treated larvae in comparison with control larvae (Figure 8B). In comparison, when chemically synthesized dsRNA were injected into *H. cunea* third instars to silence *HcAPN3* expression, a significant reduction of *HcAPN3* transcript of approximately 61% was observed 3 days after injection (*p* < 0.05, SNK test) (Figure 8A). We were unable to detect significant differences in Cry1Ab-induced mortality compared to control larvae. However, we did detect a lower percentage of larval growth inhibition after 4 days in *HcAPN3*-silenced larvae compared to control (Figure 8B).

### 2.8. Enhancement of Cry1Ab35 Toxicity by HcAPN3G and HcAPN3E

**Figure 9:**
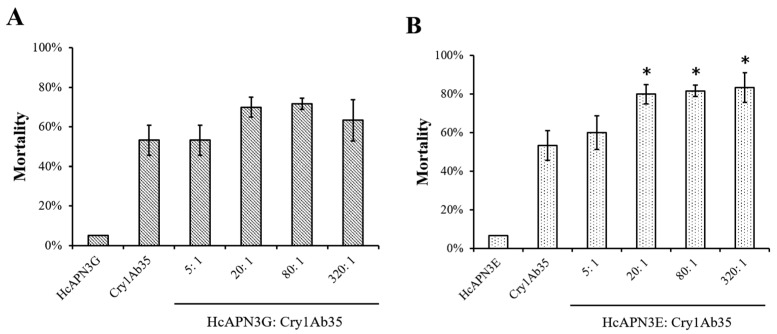
Enhancement of HcAPN3G and HcAPN3E fragment peptides to the toxicity of Cry1Ab35 toward 3 days first instars of *H. cunea* in diet overlay bioassays. Purified HcAPN3G (**A**) or HcAPN3E (**B**) peptide were mixed with Cry1Ab35 toxin at peptide:toxin mass ratios of 5:1, 20:1, 80:1, and 320:1. Control treatments included HcAPN3G or HcAPN3E peptide and Cry1Ab35 toxin alone. Each column presents data for the mean ± SEM from three replicate bioassays. An asterisk above the column indicates that the mortality of Cry1Ab35 plus fragment peptide treatment showed significant difference from Cry1Ab35 alone treatment (SNK test, *p* < 0.05).

To test the enhancement of bacterially expressed fragment peptides to the toxicity of Cry1Ab35 toxin, bioassays with fragment peptides were performed and the mortalities were measured. At the concentration of 8 μg/mL, Cry1Ab35 induced approximately 53% larval mortality. The addition of HcAPN3G peptide at peptide:toxin mass ratios of 5:1, 20:1, 80:1, and 320:1 resulted in 53%, 70%, 72%, and 63% mortality, respectively, which did not differ significantly from that of Cry1Ab35 alone (*p* > 0.05, SNK test) (Figure 9A). When HcAPN3E peptide was added to Cry1Ab35 at the ratios of 20:1, 80:1, and 320:1, the larval mortality increased to 80%, 82% and 83%, respectively, representing significant difference compared with Cry1Ab35 alone (*p* < 0.05, SNK test) (Figure 9B). In controls, insecticidal activity was rarely observed when larvae were fed with HcAPN3G or HcAPN3E peptide of highest mass alone.

Furthermore, the addition of HcAPN3E to Cry1Ab35 at the mass ratio of 20:1 resulted in an enhancement of Cry1Ab35 toxicity by 3.2-fold (LC_50_ 1.37 μg/mL, slope ± SEM, 1.37 ± 0.15; chi-square, 4.0, 95% confidence interval, 0.65–2.18 μg/mL), representing a significant synergy of Cry1Ab35 by HcAPN3E peptide. However, the mixture with HcAPN3G in the same ratio did not increase Cry1Ab35 toxicity (LC_50_ 5.25 μg/mL, slope ± SEM, 1.72 ± 0.16; chi-square, 2.56; 95% confidence interval, 3.02–10.57 μg/mL).

## 3. Discussion

In the current study, we coupled ligand blot assays with LC-MS/MS to identify Cry1Ab-binding proteins in *H. cunea* BBMV. The ligand blot approach we used has been successfully applied by others to demonstrate the Cry binding proteins in a number of insect species [14,17,30,34,35,36]. The results showed the presence of an approximately 115 kDa protein band displaying relatively higher binding intensity. Further LC-MS/MS analysis of the excised 115 kDa band indicated that APN isoforms were present in the band and probably involved in Cry1Ab binding. Moreover, several smaller protein bands (approximately 80, 72, 68, 68, 60, and 50 kDa) appearing at relatively lower intensity were also detected. Presumably, these bands were related to additional binding proteins participating in Cry1Ab toxicity. Similar binding profiles probed by Cry toxins were also detected in *M. sexta*, *O. nubilalis* and *C. suppressalis* BBMV [14,22,30].

Peptide alignment analysis of the 115 kDa band indicated that it probably corresponded to APN isoforms. The binding of Cry1A toxins to APNs in diverse classes seems to depend on sequence properties and glycosylation. Glycosylated moieties in a conserved threonine-rich region downstream of the C-terminal GPI signal sequence were believed to be an important determinant of Cry1A toxin interaction with Class 1 and Class 3 APNs [35,37,38]. Whereas for Class 4 APN in *Heliothis virescens* lacking the C-terminal threonine-rich tract, glycosylation was not important for Cry1Ac binding [39]. Thus, the absence of the C-terminal threonine-rich sequence may indicate that glycosylation sites observed in HcAPN3 were not essential for Cry1Ab binding.

The expression of HcAPN3 throughout larval development is not surprising, as this has been observed in *H. armigera* [21], *O. nubilalis* [12] and *C. suppressalis* [14]. The APN expression throughout the whole larval cycle is probably related to the need of constant food digestion to store energy and metabolites for growth and metamorphosis. As APNs are a class of digestive enzymes, the high expression levels in older larvae may be related to the high larval food intake during this phase [10].

Expression of Class 4 APNs has been generally observed in midgut tissues where APNs play an important digestive role, but the midgut is not the only tissue where they are expressed. For example, *T. ni* larvae *apn4* expression was clearly detected in Malpighian tubules [10]. A low expression of *onapn4* was also found in *O. nubilalis* Malpighian tubules [12]. *CsAPN4* transcript was also detected in hindgut and foregut of *C. suppressalis* [14]. However, expression of *bmapn4* gene in *B.mori* seemed to be midgut specific [9]. In agreement with the expression pattern observed, *HcAPN3* was strongly expressed in the midgut and hindgut tissues of *H. cunea*, then in the Malpighian tubules and fat body.

As a receptor, APN is important for the Bt pathogenic mechanism. Expression alteration of receptor APNs is correlated with insect resistance to Bt toxins, such as *T. ni* [25], *S. exigua* [23] and *Plutella xylostella* [40]. We observed that *HcAPN3* was down-regulated at 6 h and was up-regulated significantly at 12 and 24 h after Cry1Ab35 treatment. Consistent with our result, BmAPN1, −2 and −4 in *B. mori* were up-regulated at the 24 h time point after Bt infection [9]. These studies on the relationship between APNs and Cry toxins will provide a new view toward understanding the important role of APN in Cry toxicity mechanism.

Ligand blot assays detected the binding of Cry1Ab35 toxin to HcAPN3 expressed in *H. cunea* BBMV from different larval stages. Beyond our expectation, the HcAPN3 in midgut was not the principal binding protein of Cry1Ab35 toxin in the sixth instar larvae although it was expressed at a high level. We speculated that some differential modifications of HcAPN3 resulting in a decrease in its binding property to Cry1Ab35 toxin probably occurred in the sixth instar larvae. Furthermore, several smaller protein bands appearing with relatively weaker binding intensity may be considered as alternative binding proteins relevant to Cry1Ab toxicity. The 58 kDa band detected in BBMV from first and second instars presumably represented an ALP protein for its molecular size. In support of this hypothesis, ligand blot analysis using BBMV of *M. sexta* showed that Cry1Aa and Cry1Ab bound preferentially to ALP during early instars while binding to APN was observed after the third instar of larval development [17,30]. Therefore, the involvement of other binding proteins or potential receptors in Cry1Ab toxicity deserves further investigation.

RNAi has become an effective and important tool to study the function of genes in a variety of organisms. Previously, injection or feeding of dsRNA or siRNA was used to illustrate the receptor property of Cry binding proteins in diverse lepidopteran species. For example, silencing of midgut *SlAPN1* of *S. litura* by injection of dsRNA established its role as a Cry1C toxin receptor [41]. Knockdown of *HaAPN1* from *H. armigera* larvae and transfected Sf21 cells by RNAi revealed its functional interaction with Cry1Ac toxin [42]. Moreover, dietary introduction of dsRNA of *SeCad1b* in *S. exigua* suggested that it served as a putative receptor for Cry1Ca toxin [43]. Knocking down *SeALP2* from *S. exigua* by feeding siRNA revealed its role in the action mechanism of Cry2Aa toxin [44].

In this current study, the notable reduction in HcAPN3 expression did not result in significant differences in Cry1Ab35 mortality in the *HcAPN3* dsRNA-treated larvae compared to the control ones. These results indicated that multiple APN isoforms presenting in the 115 kDa binding band were probably involved in the Cry1Ab mode of action. Consistent with this hypothesis, down-regulating the expression of three DsAPN isoforms by RNAi resulted in a corresponding decrease in Cry1Ab susceptibility in *Diatraea saccharalis* larvae [45]. In *O. nubilalis*, the OnAPN1, OnAPN3a and OnAPN8 were identified as candidate receptors for Cry1Fa [22]. Moreover, RNAi knockdown of three APN isoforms (APN1, APN3a, and APN5) resulted in a decrease in Cry1Ab mortality [14]. In this study, we only focused on one APN isoform for RNAi knockdown studies to explore its functional role in Cry1Ab toxicity. Therefore, further studies should be conducted to silence other APN isoforms in *H. cunea* to demonstrate their involvements in Cry1Ab toxicity. In addition, it is also possible that additional binding proteins or receptors not identified in this work may contribute to *H. cunea* susceptibility to Cry1Ab toxin. In agreement with this hypothesis, the 80, 72, 68, 60, and 50 kDa protein bands detected in ligand blot assay (Figure 2) may represent alternative binding proteins relevant to Cry1Ab toxicity. Previous research also demonstrated that three APNs (SeAPN1, SeAPN3, and SeAPN6) and a cadherin SeCad1b were identified as putative functional Cry1Ca receptors in *S. exigua* [43,46]. Similarly, cadherin AaeCad, AaeAPN1, AaeAPN2, and ALP1 in *Aedes aegypti* were revealed to serve as candidate receptors of Cry11Aa toxin [47,48,49,50]. Therefore, the involvement of other binding proteins or potential receptors in Cry1Ab toxicity deserves further research.

The enhancement of Cry receptor fragments containing the critical toxin-binding regions to the toxicities of Cry toxins have been observed in a variety of insect species. For instance, Chen et al. reported that the CR12-MPED peptide of cadherin Bt-R1 expressed in *E. coli* functioned as a synergist of Cry1Ab toxicity against *M. sexta* [51]. Similarly, rSeCad1bp and rHaBtRp fragments differentially enhanced the negative effects of Cry1Ca and Cry1Ac on the larval mortalities and growth of *S. exigua* and *H. armigera* [43]. In addition, a truncated 70 kDa APN fragment AgAPN2tb showed a significant enhancement effect on Cry11Ba toxicity to *Anopheles gambiae* [52]. Consistent with these results, we found that the toxicity of Cry1Ab35 was enhanced by HcAPN3E fragment peptide expressed in *E. coli*. The enhancement results demonstrated the involvement of HcAPN3 in Cry1Ab35 mode of action. Furthermore, the HcAPN3E fragment peptide is proposed as the functional region for Cry1Ab binding and toxicity.

## 4. Materials and Methods

### 4.1. Insects

The *H.cunea* larvae were purchased from the Chinese Academy of Forestry Science (Beijing, China) and reared on an artificial diet under controlled temperature (26 ± 1 °C), photoperiod (16 h of light/10 h of dark), and relative humidity (75 ± 10%), without exposure to any Bt toxin.

### 4.2. Preparation of Cry1Ab35 Toxin and Antibody Preparation

Cry1Ab35 protoxin crystals from *B. thuringiensis* HD73^−^ were extracted by alkaline solubilization as described by Xue et al. [34,53]. The recombinant *B. thuringiensis* HD73^−^ strain were grown 3 days at 30 °C until sporulation and cell lysis. The crystals, spores, and debris were collected by centrifugation at 7500× *g* for 15 min, and the pellet was washed with 1 M NaCl and double-distilled water (ddH_2_O). The crystals were dissolved in 50 mM Na_2_CO_3_ (pH 9.5) containing 50 mM EDTA and 5% β-mercaptoethanol for 8 h at 4 °C and then the solution was centrifuged at 10,000× *g* for 10 min to remove any insoluble debris. The protoxin was precipitated by adding 4 M NaAc–HAc (pH 4.5) until the pH of the solution reached 4.8 (isoelectric point of Cry1Ab35), followed by incubation at 4 °C for 6 h. The protoxin pellet was collected by centrifugation at 10,000× *g* for 15 min, washed with prechilled ddH_2_O and then dissolved in 50 mM Na_2_CO_3_ (pH 10.0).

The protoxin was incubated with 1/20 (*w*/*w*) trypsin (Biosharp, Hefei, China) at 37 °C for 2 h. The activated toxin was centrifuged at 12,000× *g* for 15 min to remove insoluble debris and was then purified using a Superdex 200 column (GE HealthCare, Little Chalfont, UK). The activated Cry1Ab35 toxin (60 kDa) was analyzed by 10% sodium dodecylsulfate polyacrylamide gel electrophoresis (SDS-PAGE) and stored at −70 °C until used. Protein concentration of protoxin and activited toxin was determined by the BCA assay (Takara Bio., Dalian, China) using bovine serum albumin (BSA) as a standard. The anti-Cry1Ab35 antibodies were prepared by the Institute of biology, Hebei Academy of Sciences (Shijiazhuang, China) using the activated and purified Cry1Ab35 toxin as antigen.

### 4.3. Insect Bioassays

The susceptibility of *H. cunea* to Cry1Ab35 toxin was evaluated using diet overlay bioassays. The toxin was diluted to various concentrations in ddH_2_O and overlaid onto a 20 cm^2^ plastic tube that had been filled with artificial diet. After drying, the day 3 first instar larvae, which had been starved for 12 h, were placed in the tubes and reared under standard culture conditions. Meanwhile, the same volume of 50 mM Na_2_CO_3_ buffer was used as the control. Bioassays were repeated thrice for each treatment, and each replicate contained 20 larvae. Larval mortalities was quantified after 5 days. The 50% lethal concentration (LC_50_) values were calculated by Probit analysis.

### 4.4. Preparation of Brush Border Membrane Vesicles (BBMV)

Midguts from different larvae instars (from third to sixth) of *H.cunea* were dissected, washed in ice-cold MET buffer (300 mM mannitol, 17 mM Tris-HCl, and 5 mM EGTA, pH 7.5), then frozen in liquid nitrogen, and kept at −70 °C until used. BBMV were prepared following the MgCl_2_ differential precipitation method described by Wolfersberger et al. [36]. The midguts were homogenized in nine volumes of MET with 1 mM PMSF (Biosharp). After adding 24 mM MgCl_2_ buffer, the homogenate was gently mixed, incubated on ice for 15 min, and then centrifuged at 2500× *g* for 15 min at 4 °C. The supernatant was transferred to a new centrifuge tube and centrifuged at 30,000× *g* for 30 min at 4 °C. The pellet was resuspended in half homogenate volume of MET buffer and homogenized again. Incubation with MgCl_2_ and subsequent centrifugations were repeated as described above. The final pellet was resuspended in half-strengh MET buffer and stored at −70 °C until used. BBMV proteins from the first and second instars of *H. cunea* were prepared from whole insect bodies following procedures above. The total protein concentration was quantified as described above for toxin. Prepared BBMV samples were loaded on 10% SDS-PAGE for ligand blot assays and protein identification.

### 4.5. Ligand Blot and Protein Identification by LC-MS/MS

BBMV proteins (20 μg/well) were separated by 10% SDS-PAGE and then transferred to a polyvinylidene fluoride (PVDF) membrane (Millipore, Bedford, MA, USA). After blocking with TBS buffer (200 mM Tris-HCl, pH 7.6, 1.37 M NaCl) containing 3% BSA (Biosharp), the membrane was probed with 20 nM activited Cry1Ab35 toxin for 2 h at room temperature and subsequently washed four times for 5 min in TBS supplemented with 0.1% Tween 20 (TBST). Cry1Ab35 bound to BBMV proteins was detected by incubation of the membrane with anti-Cry1Ab35 antibodies (1:10,000 in TBS, 1 h) followed by washing with TBST and 1 h incubation with goat anti-rabbit IgG antibodies (Boster, Wuhan, China) (1:20,000 in TBS, 1 h) conjugated with alkaline phosphatase. Detection was performed by incubation of the membrane in detection buffer (100 mM Tris-HCl, pH 9.5, 100 mM NaCl, 50 mM MgCl_2_) supplemented with 2% bromochloroindolyl phosphate (BCIP)/nitro blue tetrazolium (NBT) (Biosharp) as substrates.

The corresponding band in a silver-stained gel of *H. cunea* BBMV (SDS-PAGE) was manually excised and analyzed by LCMS/MS at BGI Co., Ltd. (Wuhan, China). All LCMS/MS peptide spectra were processed and searched against the NCBInr protein database by the Mascot search engine (2.3.02 version) using a peptide mass tolerance of 20 ppm, a fragment mass tolerance of 0.1 Da and one missed cleavage. Cys carbamidomethylation was used as a fixed modification whereas Met oxidation and Asn and Gln deamidation were used as variable modifications. Proteins identified with a significant score (*p* < 0.05) were considered positive hits.

### 4.6. Cloning and Sequencing of HcAPN3 from the Midgut of H. cunea Larvae

Total RNA was extracted from the midgut of fifth instars of *H.cunea* with RNAprep pure Tissue Kit (Tiangen Co., Ltd., Beijing, China) according to the manufacturer’s instructions. The first-strand cDNA was synthesized with first-stand cDNA Synthesis Kit (Promega, Madison, WI, USA) following the manufacturer’s protocol. Primers used for PCR amplification (Table S1) were designed from two APN peptide sequences (NWGMVN and VNDVLF) identified by LC-MS/MS. The amplicon was cloned into pGEM-T Easy vector (Promega), transformed into competent *E. coli* TG1 cells (Promega) and sequenced at Sangon Biotech Co., Ltd. (Shanghai, China). Additional partial sequences were obtained by rapid amplification of cDNA ends (RACE) PCR reactions. The 5′and 3′ partial sequences were amplified using antisense and sense gene specific primers (Table S1). Specific primers (Table S1) were designed according to RACE products to obtain full-length cDNA. The resulting sequence, *HcAPN3*, was submitted to GenBank (Accession number KJ013598).

The amino acid sequence of HcAPN3 was deduced by the DNAMAN software (6.0 version). The molecular weight and isoelectric point of the predicted protein were determined using the ExPASy Compute pI/Mw tool (Available online: http://ca.expasy.org/tools/pi_tool.html). The SignalP 4.1 server (Available online: http://www.cbs.dtu.dk/services/SignalP/) was used to test for the presence of a signal peptide, and the GPI Modification Site Prediction server (Available online: http://mendel.imp.ac.at/sat/gpi/gpi_server.html) was used to predict GPI-anchor sequence and GPI anchoring sites [35,37]. The ExPASy NetOGlyc 3.1 and NetNGlyc 1.0 servers (http://www.cbs.dtu.dk/services) were used to predict potential *O*- or *N*-glycosylation sites respectively [54]. Sequence homology analyses and multiple sequence alignments were performed using NCBI/BLASTp and ClustalX 2.0, respectively. The phylogenetic tree including lepidopteran APN protein sequences available in GenBank was constructed using the neighbor-joining method with 1000 bootstrap sampling replicates with Molecular Evolutionary Genetics Analysis (MEGA) software 5.0.

### 4.7. Expression of HcAPN3 Fragment Peptides in E. coli

DNAs encoding the HcAPN3G fragment harboring the gluzincin aminopeptidase domain (16th–524th residues) and HcAPN3E fragment containing the ERAP1-like C-terminal domain (517th–952th residues) were amplified from *H. cunea* fifth instars midgut cDNA with primers listed in Table S1. The PCR products were cloned into pET-30a(+) vector and then were transformed into *E. coli* strain BL21 (DE3) for protein expression. The expression of HcAPN3G or HcAPN3E was induced by 1 mM IPTG and cultured for 6 h at 37 °C with agitation. After this period, *E. coli* cells were harvested by centrifugation at 6000× *g* for 10 min (4 °C) and then resuspended and lysed in isolation buffer (2 M urea, 10 mM Tris-HCl, 0.5 M NaCl, pH 8.0). After sonication, the inclusion bodies were isolated from the cell debris by centrifugation at 10,000× *g* for 15 min at 4 °C. This samples were separated in a 10% SDS-PAGE gel and the protein expression was confirmed using Coomassie blue staining.

The recombinant proteins expressed as inclusion bodies were isolated and solubilized in binding buffer (8 M urea, 10 mM Tris-HCl, 0.5 M NaCl, 5 mM imidazole, pH 8.0). The fragment peptides with 6 × His tag were purified with the ProteinIso Ni-NTA Resin (TransGen) according to manufacturer’s protocol and gradually dialyzed against 10 mM Tris-HCl buffer. Total protein content of each sample was determined as described above for toxin samples. The binding of Cry1Ab35 toxin to the fragment peptides were determined by ligand blot assay as described above. The anti-HcAPN3 antibodies were prepared as described above using purified HcAPN3E protein as antigen.

### 4.8. Expression of HcAPN3 in Sf9 Cells Using a Baculovirus Expression System

The complete ORF of *HcAPN3* gene were amplified with primers listed in Table S1 using PrimeSTAR HS DNA Polymerse (Takara) and cloned into the pFastBacHT vector (Invitrogen, Carlsbad, CA, USA). The recombinant plasmid (pFastBacHT-*HcAPN3*) was transformed into *E. coli* DH10Bac cells (Invitrogen) and then identified by PCR analysis. The recombinant bacmids from DH10Bac cells were purified with Hipure Plasmid Miniprep Kit (Invitrogen) according to the manufacturer’s protocol.

For expression of HcAPN3 in the *Spodoptera frugiperda* cells (Sf9), transfection was performed in a six-well format with the recombinant bacmids (1–2 mg) and Cellfectin II Reagent (Invitrogen) following manufacturer’s instructions as described by Ning et al. [40]. When the signs of infection were visually respected, Sf9 cells were collected for PCR analysis and the culture supernatants were harvested as P1 viral stock. Subsequently, the recombinant baculovirus were amplified by infection of Sf9 cells at a multiplicity of infection (MOI) of 0.05–0.1. When infection resulted in 70–80% cell mortality, the supernatants were collected as P2 viral stock. For protein expression, Sf9 cells were infected by P2 viral stock using an MOI 1–5. The infected cells were harvested at different time intervals (24, 48, 72, and 96 h) post-infection to assay for protein expression by 10% SDS-PAGE and Western blot. Transfection with the bacmid derived from pFastBac HT vector was performed as a negative control.

The expressed HcAPN3 protein containing 6 × His tag in the C-terminus was purified by affinity chromatography with the ProteinIso Ni–NTA Resin (TransGen, Beijing, China) following manufacturer’s instructions. The concentration of purified protein was measured as described above for toxin samples.

### 4.9. Detection of HcAPN3 Expression and Cry1Ab35 Binding

Western blot assay was performed to detect HcAPN3 expression. The purified HcAPN3 (20 μg/well) or BBMV (20 μg/well) samples were separated by 10% SDS-PAGE and blotted onto a PVDF membrane (Millipore). After blocked in TBS buffer supplemented with 3% BSA for 2 h at room temperature, the membrane was probed with anti-HcAPN3 antibodies (1:10,000 in TBS) for 1 h followed by washing four times for 5 min in TBST buffer. Subsequently, the membrane was incubated with goat anti-rabbit IgG antibodies (Boster) (1:20,000 in TBS) conjugated with alkaline phosphatase for 1 h. After washing as previously, the blot detection was developed in detection buffer with 2% BCIP/NBT (Biosharp) as substrate. The binding of the Cry1Ab35 toxin to HcAPN3 was determined by ligand blot assay which was carried out as described above.

### 4.10. Developmental and Tissue Expression Analysis by Semi-Quantitative PCR

Total RNA was isolated from *H.cunea* whole larvae of different developmental instars (from first to fifth) using RNAprep pure Tissue Kit (Tiangen) according to the manufacturer’s instructions. Meanwhile, RNA samples from head, foregut, midgut, hindgut, Malpighian tubules, fat body and epidermis tissues of day 3 fifth instars were prepared for tissue expression analysis. First-strand cDNA was synthesized with First-stand cDNA Synthesis Kit (Promega) following the manufacturer’s protocol. Semi-quantitative PCR reactions were carried out with GoTaq Green Master Mix (Promega) employing specific primers (Table S1). A portion of a housekeeping gene *HcActin* was used as the internal control. Each amplification was performed in triplicate. PCR products were analyzed by electrophoresis in 1% (*w*/*v*) agarose gels.

### 4.11. Induced Expression Analysis Using Quantitative Real-Time PCR (qPCR)

The *H.cunea* day 3 third instars were starved for 12 h and then were placed in plastic tubes containing artificial diet overlaid with Cry1Ab35 toxin (300 μL, 5 μg/mL). Larvae fed 50 mM Na_2_CO_3_ (pH 10.0) were used as the negative control. The midguts were collected at 0, 3, 6, 9, 12 and 24 h after treatment. The experiments were performed three independent biological replications. Extraction of total RNA from the midguts and synthsis of first-strand cDNA was done as described above. The transcript level of the *HcAPN3* in each template was estimated by qPCR method using SYBR Premix Ex Taq™II (TaKaRa) and the BIO-RAD CFX96 Touch™ Real-Time PCR Detection System (Applied Biosystems, Hercules, CA, USA). The housekeeping gene *HcActin* was selected as the internal control. The same primers (Table S1) for semi-quantitative PCR were used for qPCR analysis to amplify products less than 120 bp. Each 20 μL qPCR system contained 50 ng cDNA as the template. The following standard PCR protocol was used: denaturing at 95 °C for 30 s, followed by 40 cycles of 95 °C for 5 s, 56 °C for 30 s and 72 °C for 30 s. After amplification, the melting curves were determined by heating the sample up to 95 °C for 15 s, followed by cooling down to 50 °C for 1 min and heating the samples to 95 °C for 15 s. Each reaction was performed in triplicate. After amplification, a dissociation curve analysis was conducted to verify the absence of any nonspecific amplicons. The data were analyzed using the threshold cycle 2^−ΔΔ*C*t^ method [55].

### 4.12. RNA Interference (RNAi)

*Preparation of bacterially expressed dsRNA.* A 482 bp internal fragment of *HcAPN3* gene and a 420 bp fragment of the enhanced green fluorescent protein (*egfp*) gene (control) were obtained by PCR using the corresponding specific primers indicated in Table S1. The amplified fragments were cloned into pL4440 vector and subsequently transformed into *E. coli* HT115 (DE3) cells lacking RNase III to produce double-strand RNA (dsRNA). Cells were induced to express dsRNA by addition of 0.4 mM IPTG and incubated for 4 h at 37 °C with agitation. The total RNA of bacterial cells were extracted with RNAprep pure Cell/Bacteria Kit (Tiangen) to confirm dsRNA expression by electrophoresis on a 1% agarose gel. A large scale of bacterial cells were centrifuged at 5000× *g* for 10 min, resuspended in ddH_2_O at the ratio of 20:1 (20× concentration, the concentration of expressed dsRNA was approximately 0.5 μg/μL), and then used for bioassay.

*dsRNA ingestion assay.* Diet overlay bioassays was conducted to investigate the impact of dietary *HcAPN3* dsRNA on *H. cunea* larval sensitivity to Cry1Ab35 by a two-step feeding protocol [43]. At the first step, an aliquot of 500 μL fresh suspension from the HT115 (DE3) cells expressing *HcAPN3* dsRNA or *egfp* dsRNA (approximately 250 μg of dsRNA) or an aliquot of 500 μL ddH_2_O (negative control) was overlaid onto a 20 cm^2^ plastic tube that had been filled with artificial diet. After drying, the day 3 first instar larvae were placed in the tube. The larvae were transferred to corresponding new over-laid diets on each day, and fed for 4 days. At the second step, three larvae of each treatment were selected to extract total RNA for qPCR analysis. The other group of the larvae of each treatment were transferred to newly prepared ddH_2_O and Cry1Ab35 (10 μg/mL, equivalent to LC_70_ value)-overlaid diets. This bioassay was replicated three times, and a total of 72 larvae were used for each treatment. The larval mortalities were recorded after 5 days of treatment.

*Preparation of chemically synthesized dsRNA.* dsRNA targeting *HcAPN3* or *egfp* was chemically synthesized with T7 RiboMAX™ Express RNAi System (Promega) following the manufacturer’s instructions. The primers used to produce DNA templates with T7 promoter sequences were listed in Table S1. Large quantities of dsRNA were synthesized with 2 μg purified DNA template. Following the transcription reaction, equal volumes of complementary RNA were mixed to anneal the dsRNA and the DNA templates were removed by digestion with DNase. dsRNA were suspended in nuclease-free Water and stored at −70 °C until used. The quality and quantity of dsRNA were measured using a Fluorescent BioSpectrometer (Eppendorf, Hamburg, Germany).

*dsRNA injection assay.* Day 3 third instars of *H. cunea* were anaesthetized for 10 min on ice followed by intrahemocoelic injection of 5 μL chemically synthesized dsRNA (10 μg) between the fourth and fifth abdominal segments using a Hamilton Microsyringe. Control larvae were injected with equal volumes of ddH_2_O or *egfp* dsRNA dilutions. Following injection, larvae were kept in standard culture conditions as described above. Total RNA from the larvae was isolated at 3 day post-injection and cDNA was synthesized as described above. The transcript level of *HcAPN3* were evaluated by qPCR method. The third instars after their respective dsRNA treatments were transferred to an artificial diet containing 4 µg/mL Cry1Ab35 toxin (LC_50_ dose) for bioassays. The body weights of the larvae were measured for 4 days. There were 30 larvae tested and three times were replicated for each treatment. The percentage of growth inhibition (%GI) was calculated as %GI = (RGd/RGc) × 100%, where RGd and RGc represent the relative growth in dsRNA injection (RGd) or in the control (RGc), respectively. Relative growth (RG) was calculated as RG = [(*W*1 − *W*0)/*W*0], where *W*0 and *W*1 are the initial and final weight of the larvae, respectively.

### 4.13. Enhancement of HcAPN3 Fragment Peptides to the Toxicity of Cry1Ab35

The enhancement of HcAPN3G and HcAPN3E sensitivity to the toxicity of Cry1Ab35 in day 3 first instars of *H. cunea* was determined by the diet overlay bioassays as described above. HcAPN3G or HcAPN3E peptide was mixed with 4 μg/mL of Cry1Ab35 toxin, respectively, at different peptide: toxin mass ratios of 5:1, 20:1, 80:1, and 320:1. The maximum dose of HcAPN3G or HcAPN3E alone was used as a negative control. Larval mortalities was quantified after 5 days.

To compare the enhancement between HcAPN3G and HcAPN3E peptide, day 3 first instars of *H. cunea* were fed with Cry1Ab35, or Cry1Ab35 plus 20-fold (mass) HcAPN3G or HcAPN3E peptides. Bioassays were performed as described above. The LC_50_ values were calculated by Probit analysis.

### 4.14. Data Analysis

Larval mortality (%), growth inhibition (%), and the qPCR data were presented as means and standard errors of the mean (±SEM). One-way analysis of variance (ANOVA) with the Student-Newman–Keuls (SNK) multiple comparison test was used to determine the significance of treatment differences (overall significance level at 0.05). All the statistical analysis were performed using SPSS 22.0 for Windows (SPSS, Chicago, IL, USA).

## 5. Conclusions

In this study, we identified and characterized a putative Cry1Ab toxin-binding protein, HcAPN3, in the midgut of *H. cunea* and provided the first evidence that HcAPN3 participated in the mode of Cry1Ab action. The identification of correlated Cry1Ab binding proteins or receptors will facilitate further understanding of the Cry1Ab toxicity mechanism and help in the development of appropriate strategies for *H. cunea* control.

## Figures and Tables

**Table 1:** Candidate Cry1Ab35 binding proteins identified from the 115 kDa band from *H. cunea* BBMV by LC-MS/MS.

Protein	Protein Qscore	Protein Mass (kDa)	Unique Peptide Num	Coverage (%)	Alignment Annotation
CL698_Contig1_All	69	92.8	21	32	heat shock protein-like [*B. mori*] (XP_004927222.1)
CL2518_Contig2_All	68	104.4	22	25	aminopeptidase N-10 [*B. mori*] (AFK85026.1)
CL1273_Contig2_All	41	107.8	12	17	aminopeptidase N3 [*L. dispar*] (AAL26894.1)
CL2004_Contig1_All	39	111.6	13	18	aminopeptidase N3 [*Danaus plexippus*] (EHJ64337)
CL2587_Contig1_All	25	67.3	8	16	proton ATPase catalytic subunit A [*M. sexta*] (P31400.1)
Unigene188_All	22	104.5	7	11	aminopeptidase N-9 [*B. mori*] (AFK85025.1)
CL593_Contig3_All	9	67.0	3	8	aminopeptidase N2 [*B. mori*] (AFK85018)
CL2619_Contig1_All	8	114.2	2	6	aminopeptidase N1 [*L. dispar*] (AAD31183.1)

Binding protein corresponding to APN isoform 3 in *H. cunea* is underlined.

## Supplementary Materials

The following are available online at www.mdpi.com/1422-0067/18/12/2575/s1.

## References

[1] Gomi T. (2007). Seasonal adaptations of the fall webworm *Hyphantria cunea* (Drury) (Lepidoptera: Arctiidae) following its invasion of Japan. Ecol. Res..

[2] Zhang L.W., Kang K., Jiang S.C., Zhang Y.N., Wang T.T., Zhang J., Sun L., Yang Y.Q., Huang C.C., Jiang L.Y. (2016). Analysis of the Antennal Transcriptome and Insights into Olfactory Genes in *Hyphantria cunea* (Drury). PLoS ONE.

[3] Chen C., Wei X., Xiao H., He H., Xia Q., Xue F. (2014). Diapause induction and termination in *Hyphantria cunea* (Drury) (Lepidoptera: Arctiinae). PLoS ONE.

[4] Zhang L.W., Kang K., Liu Y.J., Zhang J., Sun L., Zhang C., Huang C.C., Jiang L.Y., Ye K.Y., Ding D.G. (2016). Evaluation of *Beauveria bassiana* isolates as potential agents for control of *Hyphantria cunea* (Lepidoptera: Arctiidae). Acta Entomol. Sin..

[5] Yang Z.Q., Wei J.R., Wang X.Y. (2006). Mass rearing and augmentative releases of the native parasitoid *Chouioia cunea* for biological control of the introduced fall webworm *Hyphantria cunea* in China. BioControl.

[6] Tang R., Zhang J.P., Zhang Z.N. (2012). Electrophysiological and behavioral responses of male fall webworm moths (*Hyphantria cunea*) to Herbivory-induced mulberry (*Morus alba*) leaf volatiles. PLoS ONE.

[7] Pigott C.R., Ellar D.J. (2007). Role of receptors in *Bacillus thuringiensis* crystal toxin activity. Microbiol. Mol. Biol. Rev..

[8] Bravo A., Sanchez J., Kouskoura T., Crickmore N. (2002). N-terminal activation is an essential early step in the mechanism of action of the *Bacillus thuringiensis* Cry1Ac insecticidal toxin. J. Biol. Chem..

[9] Lin P., Cheng T., Jin S., Jiang L., Wang C., Xia Q. (2014). Structural, evolutionary and functional analysis of APN genes in the Lepidoptera *Bombyx mori*. Gene.

[10] Wang P., Zhang X., Zhang J. (2005). Molecular characterization of four midgut aminopeptidase N isozymes from the cabbage looper, *Trichoplusia ni*. Insect Biochem. Mol. Biol..

[11] Hooper N.M. (1994). Families of zinc metalloproteases. FEBS Lett..

[12] Crava C.M., Bel Y., Lee S.F., Manachini B., Heckel D.G., Escriche B. (2010). Study of the aminopeptidase N gene family in the lepidopterans *Ostrinia nubilalis* (Hubner) and *Bombyx mori* (L.): Sequences, mapping and expression. Insect Biochem. Mol. Biol..

[13] Hughes A.L. (2014). Evolutionary diversification of aminopeptidase N in Lepidoptera by conserved clade-specific amino acid residues. Mol. Phylogen. Evol..

[14] Wang X.Y., Du L.X., Liu C.X., Gong L., Han L.Z., Peng Y.F. (2016). RNAi in the striped stem borer, *Chilo suppressalis*, establishes a functional role for aminopeptidase N in Cry1Ab intoxication. J. Invertebr. Pathol..

[15] Bravo A., Gill S.S., Soberon M. (2007). Mode of action of *Bacillus thuringiensis* Cry and Cyt toxins and their potential for insect control. Toxicon.

[16] Gomez I., Sanchez J., Miranda R., Bravo A., Soberon M. (2002). Cadherin-like receptor binding facilitates proteolytic cleavage of helix alpha-1 in domain I and oligomer pre-pore formation of *Bacillus thuringiensis* Cry1Ab toxin. FEBS Lett..

[17] Arenas I., Bravo A., Soberon M., Gomez I. (2010). Role of alkaline phosphatase from *Manduca sexta* in the mechanism of action of *Bacillus thuringiensis* Cry1Ab toxin. J. Biol. Chem..

[18] Bretschneider A., Heckel D.G., Pauchet Y. (2016). Three toxins, two receptors, one mechanism: Mode of action of Cry1A toxins from *Bacillus thuringiensis* in *Heliothis virescens*. Insect Biochem. Mol. Biol..

[19] Atsumi S., Miyamoto K., Yamamoto K., Narukawa J., Kawai S., Sezutsu H., Kobayashi I., Uchino K., Tamura T., Mita K. (2012). Single amino acid mutation in an ATP-binding cassette transporter gene causes resistance to Bt toxin Cry1Ab in the silkworm, *Bombyx mori*. Proc. Natl. Acad. Sci. USA.

[20] Knight P.J., Crickmore N., Ellar D.J. (1994). The receptor for *Bacillus thuringiensis* CrylA(c) delta-endotoxin in the brush border membrane of the lepidopteran *Manduca sexta* is aminopeptidase N. Mol. Microbiol..

[21] Angelucci C., Barrett-Wilt G.A., Hunt D.F., Akhurst R.J., East P.D., Gordon K.H., Campbell P.M. (2008). Diversity of aminopeptidases, derived from four lepidopteran gene duplications, and polycalins expressed in the midgut of *Helicoverpa armigera*: Identification of proteins binding the delta-endotoxin, Cry1Ac of *Bacillus thuringiensis*. Insect Biochem. Mol. Biol..

[22] Crava C.M., Bel Y., Jakubowska A.K., Ferre J., Escriche B. (2013). Midgut aminopeptidase N isoforms from *Ostrinia nubilalis*: Activity characterization and differential binding to Cry1Ab and Cry1Fa proteins from *Bacillus thuringiensis*. Insect Biochem. Mol. Biol..

[23] Herrero S., Gechev T., Bakker P.L., Moar W.J., de Maagd R.A. (2005). *Bacillus thuringiensis* Cry1Ca-resistant *Spodoptera exigua* lacks expression of one of four Aminopeptidase N genes. BMC Genom..

[24] Zhang S., Cheng H., Gao Y., Wang G., Liang G., Wu K. (2009). Mutation of an aminopeptidase N gene is associated with *Helicoverpa armigera* resistance to *Bacillus thuringiensis* Cry1Ac toxin. Insect Biochem. Mol. Biol..

[25] Tiewsiri K., Wang P. (2011). Differential alteration of two aminopeptidases N associated with resistance to *Bacillus thuringiensis* toxin Cry1Ac in cabbage looper. Proc. Natl. Acad. Sci. USA.

[26] Siegfried B.D., Hellmich R.L. (2012). Understanding successful resistance management: The European corn borer and Bt corn in the United States. GM Crops Food.

[27] Chen Y., Tian J.C., Shen Z.C., Peng Y.F., Hu C., Guo Y.Y., Ye G.Y. (2010). Transgenic rice plants expressing a fused protein of Cry1Ab/Vip3H has resistance to rice stem borers under laboratory and field conditions. J. Econ. Entomol..

[28] Ibiza-Palacios M.S., Ferre J., Higurashi S., Miyamoto K., Sato R., Escriche B. (2008). Selective inhibition of binding of *Bacillus thuringiensis* Cry1Ab toxin to cadherin-like and aminopeptidase proteins in brush-border membranes and dissociated epithelial cells from *Bombyx mori*. Biochem. J..

[29] Tanaka S., Miyamoto K., Noda H., Endo H., Kikuta S., Sato R. (2016). Single amino acid insertions in extracellular loop 2 of *Bombyx mori* ABCC2 disrupt its receptor function for *Bacillus thuringiensis* Cry1Ab and Cry1Ac but not Cry1Aa toxins. Peptides.

[30] Flores-Escobar B., Rodriguez-Magadan H., Bravo A., Soberon M., Gomez I. (2013). Differential role of *Manduca sexta* aminopeptidase-N and alkaline phosphatase in the mode of action of Cry1Aa, Cry1Ab, and Cry1Ac toxins from *Bacillus thuringiensis*. Appl. Environ. Microbiol..

[31] Flannagan R.D., Yu C.G., Mathis J.P., Meyer T.E., Shi X., Siqueira H.A., Siegfried B.D. (2005). Identification, cloning and expression of a Cry1Ab cadherin receptor from European corn borer, *Ostrinia nubilalis* (Hubner) (Lepidoptera: Crambidae). Insect Biochem. Mol. Biol..

[32] Ferre J., Van Rie J. (2002). Biochemistry and genetics of insect resistance to *Bacillus thuringiensis*. Annu. Rev. Entomol..

[33] Moore R.E., Young M.K., Lee T.D. (2002). Qscore: An algorithm for evaluating SEQUEST database search results. J. Am. Soc. Mass Spectrom..

[34] Luo K., Banks D., Adang M.J. (1999). Toxicity, binding, and permeability analyses of four *Bacillus thuringiensis* Cry1 delta-endotoxins using brush border membrane vesicles of *Spodoptera exigua* and *Spodoptera frugiperda*. Appl. Environ. Microbiol..

[35] Wolfersberger M.G. (1993). Preparation and partial characterization of amino acid transporting brush border membrane vesicles from the larval midgut of the gypsy moth (*Lymantria dispar*). Arch. Insect Biochem. Physiol..

[36] Wolfersberger M., Luethy P., Maurer A., Parenti P., Sacchi F.V., Glordana B., Hanozet G.M. (1987). Preparation and partial characterization of amino acid transporting brush border membrane vesicles from the larval midgut of the cabbage butterfly (*Pieris brassicae*). Comp. Biochem. Physiol..

[37] Eisenhaber B., Bork P., Eisenhaber F. (2001). Post-translational GPI lipid anchor modification of proteins in kingdoms of life: Analysis of protein sequence data from complete genomes. Protein Eng..

[38] Hoogland C., Mostaguir K., Appel R.D., Lisacek F. (2008). The World-2DPAGE Constellation to promote and publish gel-based proteomics data through the ExPASy server. J. Proteom..

[39] Banks D.J., Jurat-Fuentes J.L., Dean D.H., Adang M.J. (2001). *Bacillus thuringiensis* Cry1Ac and Cry1Fa delta-endotoxin binding to a novel 110 kDa aminopeptidase in *Heliothis virescens* is not *N*-acetylgalactosamine mediated. Insect Biochem. Mol. Biol..

[40] Ning C., Wu K., Liu C., Gao Y., Jurat-Fuentes J.L., Gao X. (2010). Characterization of a Cry1Ac toxin-binding alkaline phosphatase in the midgut from *Helicoverpa armigera* (Hubner) larvae. J. Insect Physiol..

[41] Rajagopal R., Sivakumar S., Agrawal N., Malhotra P., Bhatnagar R.K. (2002). Silencing of midgut aminopeptidase N of *Spodoptera litura* by double-stranded RNA establishes its role as *Bacillus thuringiensis* toxin receptor. J. Biol. Chem..

[42] Sivakumar S., Rajagopal R., Venkatesh G.R., Srivastava A., Bhatnagar R.K. (2007). Knockdown of aminopeptidase-N from *Helicoverpa armigera* larvae and in transfected Sf21 cells by RNA interference reveals its functional interaction with *Bacillus thuringiensis* insecticidal protein Cry1Ac. J. Biol. Chem..

[43] Ren X.L., Chen R.R., Zhang Y., Ma Y., Cui J.J., Han Z.J., Mu L.L., Li G.Q. (2013). A *Spodoptera exigua* cadherin serves as a putative receptor for *Bacillus thuringiensis* Cry1Ca toxin and shows differential enhancement of Cry1Ca and Cry1Ac toxicity. Appl. Environ. Microbiol..

[44] Yuan X., Zhao M., Wei J., Zhang W., Wang B., Myint Khaing M., Liang G. (2017). New insights on the role of alkaline phosphatase 2 from *Spodoptera exigua* (Hubner) in the action mechanism of Bt toxin Cry2Aa. J. Insect Physiol..

[45] Yang Y., Zhu Y.C., Ottea J., Husseneder C., Leonard B.R., Abel C., Huang F. (2010). Molecular characterization and RNA interference of three midgut aminopeptidase N isozymes from *Bacillus thuringiensis*-susceptible and -resistant strains of sugarcane borer, *Diatraea saccharalis*. Insect Biochem. Mol. Biol..

[46] Ren X.L., Ma Y., Cui J.J., Li G.Q. (2014). RNA interference-mediated knockdown of three putative aminopeptidases N affects susceptibility of *Spodoptera exigua* larvae to *Bacillus thuringiensis* Cry1Ca. J. Insect Physiol..

[47] Chen J., Aimanova K.G., Fernandez L.E., Bravo A., Soberon M., Gill S.S. (2009). *Aedes aegypti* cadherin serves as a putative receptor of the Cry11Aa toxin from *Bacillus thuringiensis* subsp. israelensis. Biochem. J..

[48] Jimenez A.I., Reyes E.Z., Cancino-Rodezno A., Bedoya-Perez L.P., Caballero-Flores G.G., Muriel-Millan L.F., Likitvivatanavong S., Gill S.S., Bravo A., Soberon M. (2012). *Aedes aegypti* alkaline phosphatase ALP1 is a functional receptor of *Bacillus thuringiensis* Cry4Ba and Cry11Aa toxins. Insect Biochem. Mol. Biol..

[49] Chen J., Aimanova K.G., Pan S., Gill S.S. (2009). Identification and characterization of *Aedes aegypti* aminopeptidase N as a putative receptor of *Bacillus thuringiensis* Cry11A toxin. Insect Biochem. Mol. Biol..

[50] Chen J., Likitvivatanavong S., Aimanova K.G., Gill S.S. (2013). A 104 kDa *Aedes aegypti* aminopeptidase N is a putative receptor for the Cry11Aa toxin from *Bacillus thuringiensis* subsp. *israelensis*. Insect Biochem. Mol. Biol..

[51] Chen J., Hua G., Jurat-Fuentes J.L., Abdullah M.A., Adang M.J. (2007). Synergism of *Bacillus thuringiensis* toxins by a fragment of a toxin-binding cadherin. Proc. Natl. Acad. Sci. USA.

[52] Zhang R., Hua G., Urbauer J.L., Adang M.J. (2010). Synergistic and inhibitory effects of aminopeptidase peptides on *Bacillus thuringiensis* Cry11Ba toxicity in the mosquito *Anopheles gambiae*. Biochemistry.

[53] Xue J., Liang G.M., Crickmore N., Li H.T., He K.L., Song F.P., Feng X., Huang D.F., Zhang J. (2008). Cloning and characterization of a novel Cry1A toxin from *Bacillus thuringiensis* with high toxicity to the Asian corn borer and other lepidopteran insects. FEMS Microbiol. Lett..

[54] Gasteiger E., Gattiker A., Hoogland C., Ivanyi I., Appel R.D., Bairoch A. (2003). ExPASy: The proteomics server for in-depth protein knowledge and analysis. Nucleic Acids Res..

[55] Pfaffl M.W. (2001). A new mathematical model for relative quantification in real-time RT-PCR. Nucleic Acids Res..

