# Supplementary material for: Identification and Characterization of Hyphantria cunea Aminopeptidase N as a Binding Protein of Bacillus thuringiensis Cry1Ab35 Toxin
Source: Int J Mol Sci. 2017 Nov 30;18(12):2575. doi: 10.3390/ijms18122575 (PMC5751178; doi:10.3390/ijms18122575)
Supplement: Supplementary file 1 [file ijms-18-02575-s001.pdf]

Table S1. Primers used for gene amplication and expression, dsRNA synthesis and qPCR analysis of *HcAPN3*.

Primer	Forward sequence (5′–3′)	Reverse sequence (5′–3′)
Reverse transcription PCR	GAACTGGGGAATGGTTAACT	AGAACAAAACGTCGTTGAC
5′ RACE		CGCTATACAATCTCCGGGGTTAGGCCAGCTG
3′ RACE	CTAGTGGAGAAGTGGGCAGTGATTGAAGGGAGA	
Cloning and expression of full length of HcAPN3		
	AAATATGCGGCCGCTACCAACGCCATGATTCTATCGATC	CCGCTCGAGATGGAACAACTGGGCGAATAGTACTGC
Semi-quantitative PCR and qPCR		
HcAPN3	AATGAGAATCCCGTAGACCG	TAGGCTCGTCAAAGCAAGG
HcActin	CTACCTCACGCCATTCTC	AGCTTCTCCTTGATGTCAC
RNAi		
Preparation of bacterially expressed dsRNA		
HcAPN3	AACTGCAGAGTCGGTGTATCCTCACTTTATG	ACGCGTCGACAATGCTTTTCTGGCGTTTC
egfp	AACTGCAGCCACAAGTTCAGCGTGTCCG	ACGCGTCGACAGTTCACCTTGATGCCGTTCT
Preparation of chemically synthesized dsRNA		
HcAPN3-template	TAATACGACTCACTATAGGAGTCGGTGTATCCTCACTTTATG	TAATACGACTCACTATAGGAATGCTTTTCTGGCGTTTC
egfp-template	TAATACGACTCACTATAGGCCACAAGTTCAGCGTGTCCG	TAATACGACTCACTATAGGAGTTCACCTTGATGCCGTTCT
HcAPN3	AGTCGGTGTATCCTCACTTTATG	AATGCTTTTCTGGCGTTTC
egfp	CCACAAGTTCAGCGTGTCCG	AGTTCACCTTGATGCCGTTCT
Expression of *HcAPN3G*	AAATATGCGGCCGCGCCTTCCCCGATGAAGACTACAGGT	CCGCTCGAGTACAGGATCCAGGGAAGCAAAAACAA
Expression of *HcAPN3E*	AAATATGCGGCCGCGTTTTTGCTTCCCTGGATCCTGTA	CCGCTCGAGATGGAACAACTGGGCGAATAGTACTGC

Restrictions sites are underlined.
